# Arenobufagin Promoted Oxidative Stress-Associated Mitochondrial Pathway Apoptosis in A549 Non-Small-Cell Lung Cancer Cell Line

**DOI:** 10.1155/2020/8909171

**Published:** 2020-04-22

**Authors:** Jun Kan, Haifu Huang, Zhangyu Jiang, Ruisheng Zhou, Shasha Bai, Caijie Liao, Jiancong Chen, Jun Dong, Yunlong Zhang, Jingzhi Zhang, Rong Zhang, Daihan Zhou, Enxin Zhang

**Affiliations:** ^1^Department of Oncology, The First Affiliated Hospital of Guangzhou University of Chinese Medicine, Guangzhou 510006, China; ^2^Shenzhen Hospital (Futian) of Guangzhou University of Chinese Medicine, Shenzhen 518000, China; ^3^Guangzhou University of Chinese Medicine, Guangzhou 510006, China; ^4^Department of Integrated Therapy in Oncology, Sun Yat-sen University Cancer Center, State Key Laboratory of Oncology in South China, Collaborative Innovation Center for Cancer Medicine, Guangzhou 510060, China; ^5^Key Laboratory of Neuroscience, School of Basic Medical Sciences, Guangzhou Medical University, Guangzhou 511436, China; ^6^The Second Affiliated Hospital, Guangzhou Medical University, Guangzhou 510260, China; ^7^Institute of Clinical Pharmacology, Guangzhou University of Chinese Medicine, Guangzhou 510006, China

## Abstract

Arenobufagin (ARE) has demonstrated potent anticancer activity in various types of tumor, but the role and mechanism of ARE for lung cancer remain unclear. Oxidative stress exists under normal conditions and is an inevitable state in the body. A variety of noxious stimuli can break the equilibrium state of oxidative stress and promote apoptosis. Here, we used a CCK-8 assay to examine cell viability. We determined oxidative stress damage by measuring levels of intracellular ROS and levels of GSH, SOD, and MDA. Annexin V-FITC/PI double staining assay, as well as the Hoechst 33258 staining, was used to detect ARE-induced apoptosis in A549 cell. Evaluation of the expression level of the specified molecule was indicated by Western blot and qRT-PCR. Loss of function experiment was carried out using NAC pretreatment. The experimental results show that ARE significantly declines in the viability of A549 cells and increases the apoptosis rate of A549 cells. As reflected in cell morphology, the A549 cells showed features of shrinkage and had incompletely packed membranes; the same phenomenon is manifested in Hoechst 33258 staining. Following ARE treatment, the ROS level in A549 cells was rising in a concentration-dependent manner, and so were MDA and GSH levels, while the SOD level was decreasing. Moreover, we found that ARE can decrease mitochondrial membrane potential (MMP), and a cascade of apoptotic processes can be triggered by decreased MMP. Importantly, we found significant changes in protein expression levels and mRNA levels of apoptosis-related proteins. Furthermore, when we used NAC to restrain oxidative stress, the expression levels of apoptosis-related proteins have also changed accordingly. Our data demonstrate that apoptosis in the non-small-cell lung cancer (NSCLC) cell line A549 is caused by oxidative stress due to ARE. Our research also shows that ARE may have the potential to become a targeted therapeutic for the treatment of NSCLC in the future.

## 1. Introduction

Lung cancer (LC) is one of the most common malignancies from poles to poles. According to the results of the American Cancer Society in 2018, lung cancer accounts for 11.6% of total cancers, and lung cancer mortality accounts for 18.4% of total cancer deaths [1]. According to its biological characteristics, lung cancer can be segmented into two types, small cell lung cancer (SCLC) and non-small-cell lung cancer (NSCLC). Among them, the percentage of NSCLC in all lung cancer incidences is 80.4% [2]. The treatment of NSCLC mainly includes surgery, radiotherapy and chemotherapy, targeted therapy, and immunotherapy. Although the treatment is changing with each passing day, the 5-year survival rate is still not ideal [3]. Therefore, antineoplastic agents for NSCLC patients are urgently needed. Oxidative stress exists under normal conditions and is an inevitable state in the body. A variety of noxious stimuli can break the equilibrium state of oxidative stress and promote apoptosis [4]. Many diseases, such as Parkinson's disease and cancer, are associated with oxidative stress [5]. One of the main free radical scavenging enzymes in the body is superoxide dismutase (SOD). Malondialdehyde (MDA) is produced by catalytic cracking of lipid peroxide in the presence of metal ions and caused by a decrease in SOD activity. MDA is toxic to cells and can lead to intramolecular and intermolecular proteins cross-linking to induce apoptosis [6]. In oxidative stress-mediated apoptosis, caspase activation and changes in Bcl-2-related proteins occur repeatedly, which has an important influence on the process of apoptosis [7]. The endogenous apoptotic pathway involves the suppression of the Bcl-2-encoding gene and binding of the proapoptotic protein Bax to the mitochondrial membrane [8]. By repressing Bax activation and its secondary mitochondrial membrane translocation, anti-Bcl-2 apoptotic proteins play a role in protecting mitochondria and prevent Bax from destruction of mitochondrial outer membrane completeness and suppression of caspase precursor activation, thereby resulting in cells apoptosis [9].

**Figure 1:**
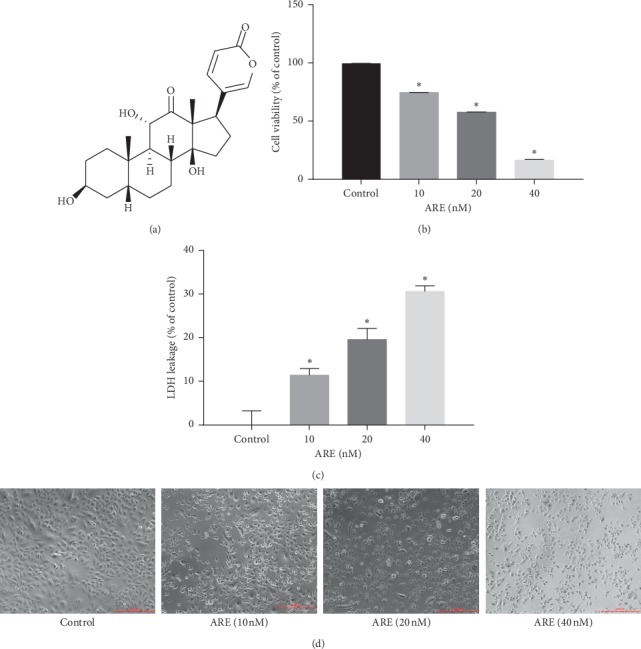
ARE-induced injury in A549 cell. (a) Chemical structure of ARE. (b) A549 cells were handled with ARE at a concentration of 10–40 nM for 24 h, and CCK-8 assay was used to detect the cell activity. (c) ARE treatment increased the LDH release in A549 cells. (d) Morphological changes of A549 cells were induced by ARE. The results are expressed as mean ± SD (*n* = 3). ^*∗*^*P* < 0.05 versus the control group.

There is growing interest in anticancer agents for natural products used in traditional Chinese medicine [10]. Arenobufagin (ARE, structure shown in Figure 1(a)), one of the effective constituents of toad venom, is a traditional Chinese medicine obtained from the skin and parotid venom glands of *Bufo bufo gargarizans* Cantor or *Bufo melanostictus* Schneider [11]. Anyway, a lot of studies have demonstrated its broad-spectrum antitumor activities in cancers such as breast cancer, pancreatic carcinoma, and liver cancer [12–14]. We previously found that ARE can induce liver cancer cell apoptosis and autophagy through PI3K/Akt/mTOR signal routing [14]; induce cell cycle arrest and apoptosis in human cervical cancer HeLa cells [15]; have anticancer effect on human esophageal squamous cell carcinoma (its mechanism of exerting anticancer efficacy may be activation of cysteine-containing aspartate proteolytic enzyme (caspase) by endogenous and exogenous pathways); promote apoptosis of esophageal cancer cells by enhancing caspase phosphorylation and activating p53 signaling [16]; promote apoptosis of human glioblastoma U-87 cells by inhibiting p38MAPK signaling pathway [17]; and inhibit epithelial-mesenchymal conversion by going down the *β*-catenin pathway, consequently repressing motility and invasiveness of prostate cancer PC3 cells [18]. Nevertheless, the role and mechanisms of ARE on NSCLC remain unclear.

In this study, we scientifically appraised the anticancerous function of ARE on A549 NSCLC cell line. Our results indicate that ARE remarkably inhibits the growth of A549 cells and induces apoptosis. What is important is that we report a new discovery that activation of the oxidative stress pathway is critical in the apoptosis induced by ARE. The above results indicate that ARE may be a promising drug for NSCLC patients.

## 2. Materials and Methods

### 2.1. Materials and Reagents

ARE with a molecular formula of C_24_H_32_O_6_ and a molecular weight of 416.51 was obtained from MedChemExpress (≥99.42% purity, MCE, USA). The Cell-Counting Kit-8 (CCK-8) assay was purchased from Dojindo (Japan). The antioxidant agent (N-acetyl-L-cysteine, NAC) and dimethyl sulfoxide (DMSO) were procured from Sigma (St. Louis, MO, USA). Glutathione (GSH), malondialdehyde (MDA), and superoxide dismutase (SOD) were obtained from Nanjing Jiancheng Co. (Nanjing, China). BCA protein assay kits were from Kaiji Co. (Jiangsu, China). Annexin V fluorescein-isothiocyanate (FITC)/propidium iodide (PI) apoptosis detection kits were purchased from BestBio Institute of Biotechnology (Shanghai, China). Primary antibodies against Bax, Bcl-2, and GAPDH were obtained from Affinity (Affinity Biosciences, OH, USA). Primary antibodies against caspase-3 and caspase-9 were obtained from Abcam (Cambridge Science Park, UK). Anti-rabbit IgG and anti-mouse IgG, HRP-linked antibody, were from Beijing Dingguo Changsheng Biotechnology Co. Ltd. (Beijing, China). Enhanced chemiluminescence (ECL) kits were purchased from Affinity (Affinity Biosciences, OH, USA).

### 2.2. Cell Lines and Cell Culture

A549 cells were cultured in DMEM-high glucose (Gibco, Grand Island, NY, USA) containing 10% fetal bovine serum (FBS) and 1% penicillin/streptomycin (Gibco, Grand Island, NY, USA) in a CO_2_ humidified incubator (Sanyo, Japan) at 37°C with 5% CO_2_. Fresh media should be replaced every three days during cell culture. ARE was dissolved in DMSO, stored at 20°C, and then defrosted when needed (DMSO <0.01%).

### 2.3. Cell Viability Assay

A549 cells were digested by trypsinization and then digested with twice complete medium to prepare a cell suspension. After cell counting, the cell concentration was adjusted to 8 × 10^4^ cells/ml to 100 *μ*L/well (that is, 8000 cells per well); cells were inoculated into a 96-well plate, and the peripheral wells were filled with 100 *μ*L of PBS buffer and cultured in a 37°C, 5% CO_2_ incubator. The original medium was removed; 100 *μ*L of the medium with the ARE concentrations of 10 nM, 20 nM, and 40 nM were administrated after the cells in each well of the 96-well plate were attached; and the control group (medium and cells) and the blank group (medium, no cells) were determined. There are three time gradients of 12 h, 24 h, and 48 h. Five replicate wells were set in each group and placed in a 37°C, 5% CO_2_ incubator for further culture. After 12 h, 24 h, and 48 h of A549 cells drug intervention, the original medium was removed and 100 *μ*L medium (containing 10 *μ*L CCK-8 solution) was put into each well and then continued to be cultured in the incubator. After 1 hour, the culture was terminated. The experimental results were recorded after measuring the absorbance at 450 nm with a microplate reader.

### 2.4. Measuring the Contents of Intracellular MDA, GSH, and SOD

A549 cells (4 × 10^5^ cells/well) were inoculated into 6-well plates, then cells were collected after 24 hours of drug exposure, and the contents of GSH, MDA, and SOD were measured. According to the instructions of the manufacturer, GSH, MDA, and SOD detection kits (Nanjing Jiancheng Bioengineering Institute, Nanjing, China) were used to determine the contents of GSH, MDA, and SOD.

### 2.5. Detection of Intracellular ROS Level

Based on the instructions (Nanjing Jiancheng Bioengineering Institute, Nanjing, China), DCFH-DA was used as a label to test the production of intracellular ROS. In a word, the fluorescence microscope (Leica, Germany) was used to observe the intensity of green fluorescence in A549 cells after 24 h of ARE administration, and the cells were analyzed for their reactive oxygen species by flow cytometry using a 485 nm excitation filter and a 525 nm emission filter.

### 2.6. Hoechst 33258 Staining

A549 cells were treated with ARE (10–40 nM) for 24 h, then washed twice with cold PBS, and then incubated with Hoechst 33258 fluorescent dye (10 *μ*g/ml) for 10 minutes at room temperature. Cellular fluorescence was then observed using a fluorescence microscope (Leica, Germany).

### 2.7. Observation of Mitochondrial Membrane Potential (MMP)

The MMP after treatment of A549 cells with ARE was evaluated by JC-1. In a word, A549 cells were treated with ARE (10–40 nM) for 24 h, then washed twice with cold PBS, and incubated with JC-1 for 20 min at 37°C in a 5% CO_2_ atmosphere. Finally, the cells were washed twice with configured JC-1 dyeing buffer (1x), each time for 5 min. The fluorescence microscope (Leica, Germany) was used to evaluate the A549 cells stained with JC-1.

### 2.8. Cell Apoptosis Test

The apoptosis of A549 cells treated with ARE of 24 h was detected according to the instructions of the FITC-Annexin V/propidium iodide (PI) apoptosis detection kit (BestBio, Shanghai, China). A549 cells were disposed as described above. Thereafter, A549 cells were collected by centrifugation at 1000 × *g* for 5 min and then washed with cold PBS two times to adjust to a concentration of 1 × 10^6^ cells/ml. Afterwards, the cells of each group were adjusted to approximately 1 × 10^6^ cells, resuspended in 400 *μ*L binding buffer, then incubated with 5 *μ*L Annexin V-FITC for 10 min, and then resuspended with 5 *μ*L PI at room temperature for 5 min. The samples were detected using an FACS Canto II flow cytometer (Becton Dickinson, USA) within 1 h.

### 2.9. NAC Pretreatment

A549 cells were pretreated with N-acetyl-L-cysteine (NAC) to restrain oxidative stress. In short, A549 cells were cultured in 10 cm^2^ cell culture dishes for 24 hours and pretreated for 1 hour to prevent ROS generation. Then cultivated A549 cells were collected for Western blot analysis.

### 2.10. RNA Extraction and Quantitative Real-Time PCR (qRT-PCR)

After being cultured for 24 hours in a 60 mm culture dish, the A549 cells were treated with 10, 20, and 40 nM ARE for 24 hours, and then the old medium was drained and washed twice with cold PSB. Total RNA in cells was extracted with Trizol (Takara, Japan), and then 500 ng of total RNA samples were reverse-transcribed into cDNA by using PrimeScript™ RT Master Mix (Takara, Japan) after measuring the concentration of each group of RNA. Each sample was then subjected to real-time PCR using the double-stranded DNA dye SYBR Premix Ex Taq™ II kit (Takara, Japan) according to the manufacturer's structure of Bax, Bcl-2, caspase-9, caspase-3, and GAPDH. The reaction conditions were 95°C for 35 s, annealing for 30 s at 60°C, and 40 cycles. The internal comparison was GAPDH (Shenggong Biotech, China). The primer information for this experiment is as follows:

  Bax forward, 5′-GATGCGACCAAGAAGCTAAG-3′;
  Bax reverse, 5′-CACGGCGGCAATCATCCTCTG-3′;
  Bcl-2 forward, 5′-GTGGATGACTGAGTACCTGAACCG-3′;
  Bcl-2 reverse, 5′-AGAGTCTTCAGAGACAGCCAGGAG-3′;
  caspase-9 forward, 5′-CTGCTGCGTGGTGGTCATTCTC-3′;
  caspase-9 reverse, 5′-CACAATCTTCTCGACCGACACAGG-3′;
  caspase-3 forward, 5′-TGGAAGCGAATCAATGGACTCTGG-3′;
  caspase-3 reverse, 5′-CCAGACCGAGATGTCATTCCAGTG-3′;
  GAPDH forward, 5′-GCCACAGTCAAGGCTGAGAATG-3′;
  GAPDH reverse, 5′-ATGGTGGTGAAGACGCCAGTA-3′.

RT-qPCR analysis was performed using triplet repeat PCR. Data were analyzed by the 2^−ΔΔCt^ method.

### 2.11. Western Blot Analysis

Cultivated A549 cells were collected, washed three times with cold PBS, and lysed with RIPA Lysis Buffer (Strong) (CWBIO, Beijing, China) to prepare the total protein fractions. The total protein extract (30 *μ*g) of the cells was isolated by using SDS-PAGE at 80 V for 2 h at indoor temperature and electrotransferred onto PVDF membranes (0.45 *μ*M) at 300 mA for 80 min at ice. The membranes were washed four times with TBST (1x), each for 5 min, sealed with TBST (1x) diluted 5% skim milk powder for 2 h at room temperature, and washed with TBST (1x) twice, each time for 1 min. The membranes were then incubated with primary antibodies against Bcl-2, cleaved caspase-3 at 1 : 1000 dilution, caspase-9 at 1 : 2000 dilution, *β*-Actin at 1 : 3000 dilution, GAPDH at 1 : 5000 dilution; next incubated with HRP-linked anti-IgG for 80 min at room temperature; and then washed four times as described above. In the meantime, the control was GAPDH protein. The chemiluminescent of various proteins were then analyzed using Image Lab™ software on ChemiDoc XRS^+^ (Bio-Rad, USA).

### 2.12. Statistical Analysis

All experiments were performed three times. All data were expressed as mean ± SD (standard deviation) and were analyzed using one-way ANOVA. SPSS 17.0 software (version 17.0) was used for all statistical analyses. Statistically significant data is represented by *P* values <0.05.

## 3. Results

### 3.1. ARE-Induced A549 Cell Damage

Figure 1(a) is the structure graph of ARE. First, we investigated the effect of ARE on the viability and proliferation of A549 cells. The viability and proliferation of A549 cells treated with 10–40 nM ARE for 24 hours showed significant changes, as shown in Figure 1(b). Cell viability was reduced to approximately 16.56% based on CCK-8 assay at 40 nM ARE. ARE dose-dependently reduced cell viability (Figure 1(b)). LDH leakage is thought to be an indication of cell damage which can suppress plasma membrane destruction that was remarkably increased after ARE administration (Figure 1(c)). Furthermore, morphological changes of A549 cells in different experimental groups were observed by optical microscope. As shown in Figure 1(d), control cells appeared to have a dense membrane and a natural cell shape and structure. However, the A549 cells exposed to ARE treatment showed features of shrinkage and had incompletely packed membranes.

### 3.2. ARE-Induced A549 Cell Apoptosis

**Figure 2:**
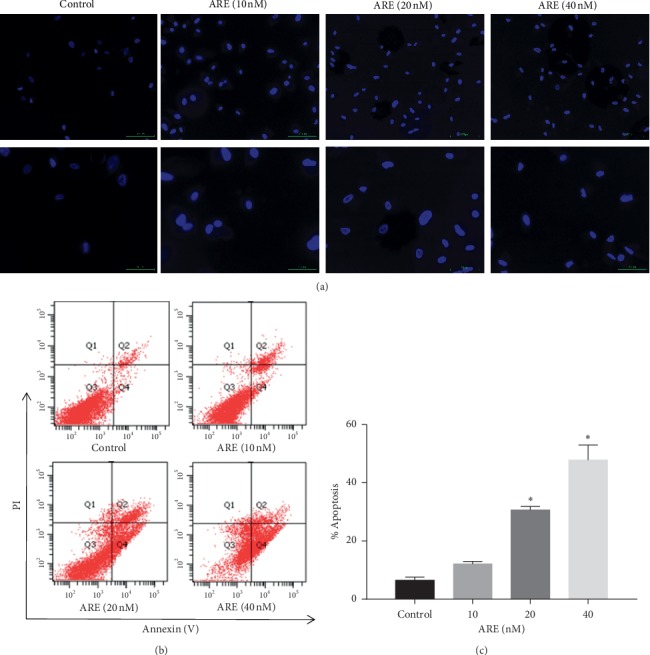
ARE-induced A549 cell apoptosis. A549 cells were pretreated with ARE at concentrations of 10, 20, and 40 nM for 24 h. (a) Hoechst 33258 staining revealed ARE-induced chromatin contraction in A549 cells in each group. Scale bars: 100 *μ*m; magnification: 50 *μ*m. (b), (c) Flow cytometry was used to detect apoptotic cells: (b) the raw flow cytometry figures; (c) the apoptosis rates. The results are expressed as mean ± SD (*n* = 3). ^*∗*^*P* < 0.05 versus the control group.

We also tried to confirm whether the cytotoxic effect of ARE is related to apoptosis. We detected apoptosis in A549 cells using Hoechst 33258 assay and flow cytometry. The results of Hoechst 33258 assay showed brighter nuclei staining and condensation of nuclear chromatin and nuclear breakdown, indicating that the ARE treatment group had more apoptosis rate than the control group (Figure 2(a)). Moreover, flow cytometry was used to support the accelerated effect of ARE on apoptosis of A549 cells (Figure 2(b)). The data showed that the apoptotic rate of A549 cells after ARE administration was higher than that of the control group, showing a concentration dependence. At 40 nM ARE, the number of apoptotic cells increased to 48.00%. The apoptosis rate of A549 cells increased after they were exposed to ARE (Figure 2(c)).

### 3.3. Mitochondrial Pathway Performs Key Functions in ARE-Induced Apoptotic Cell Death

**Figure 3:**
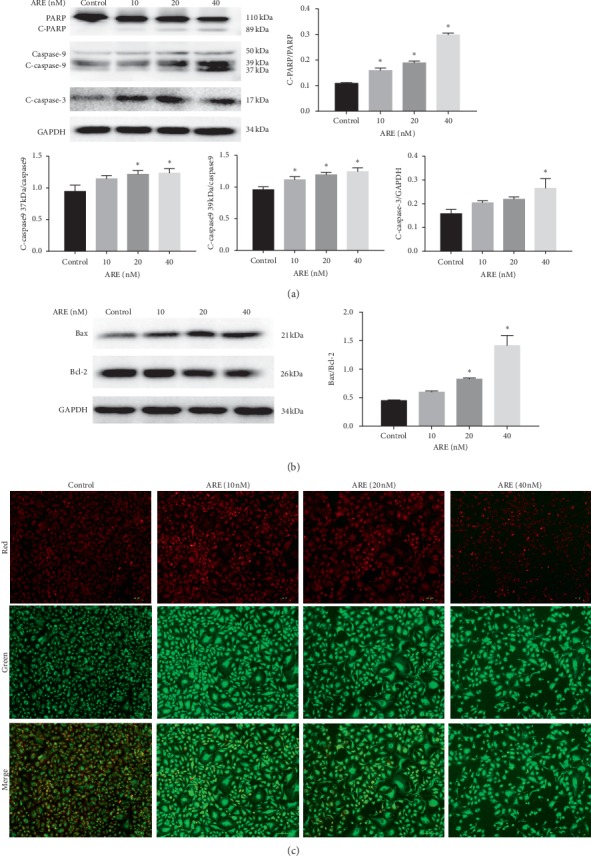
The effect of ARE on proapoptotic protein expression and MMP. A549 cells were treated with ARE (10–40 nM) for 24 h. (a) ARE-induced cleaved PARP, cleaved caspase-3, and caspase-9 expression. (b) ARE increased Bax expression but reduced Bcl-2 expression. (c) A549 cells are administered 24 hours after ARE administration; they were stained with JC-1. After that the mitochondrial membrane potential was observed under the fluorescence microscope. Scale bars: 100 μm. The results are expressed as mean ± SD (*n* = 3). ^*∗*^*P* < 0.05 versus the control group.

To examine whether ARE can adjust the expression of apoptosis proteins, Western blotting was used to assess the expression of caspase-3/9 and PARP after 24 hours of ARE treatment at 10, 20, and 40 nM. There was a dose-dependent increase in cleavage of caspase-3/9 in the ARE-treated groups compared with the control (Figure 3(a)). At the same time, the cleavage of PARP expression presented a similar trend (Figure 3(a)). These consequences indicated that apoptosis was induced by ARE in a dose-dependent manner. A cascade of apoptotic processes can be triggered by the decline of MMP. To determine whether ARE-induced apoptosis was associated with MMP pathway in A549 cells, JC-1 staining was used to estimate MMP. As presented in Figure 3(c), normal mitochondria showed red fluorescence after JC-1 staining. A549 cells exposed to ARE increased green fluorescence, indicating a decrease in MMP and a decrease in red/green fluorescence ratio, which was relevant to apoptosis. The MMP loss of A549 cells increased with A549 treatment at 10, 20, and 40 nM compared with control group (Figure 3(c)). The loss of MMP is an immediate response to the impact of ARE on mitochondrial function, which could then activate the caspase family, thus causing apoptosis of A549 cells. MMP is mainly regulated by antiapoptotic Bcl-2 and proapoptotic Bax proteins. After exposure to ARE, Bax levels were strikingly increased, while Bcl-2 levels were strikingly reduced (Figure 3(b)). All these measurements clarified that ARE-induced cell damage might be ascribed to mitochondria apoptosis pathway.

### 3.4. Changes of mRNA Expression Levels of Parameters Associated with Mitochondria Apoptosis Pathway in the A549 Cells

**Figure 4:**
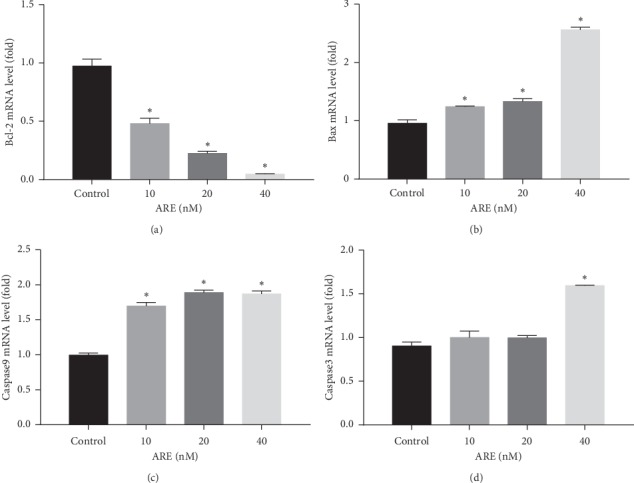
Effect of ARE on the expression of apoptosis-related genes in A549 cells. A549 cells were treated with ARE (10–40 nM) for 24 h. The mRNA levels of Bcl-2, Bax, caspase-9, and caspase-3 were measured by real-time PCR (Figures 4(a)–4(d)). The results are expressed as mean ± SD (*n* = 3). ^*∗*^*P* < 0.05 versus the control group.

The results of qRT-PCR showed that ARE can upregulate the transcription level of A549 cells by the mitochondrial apoptosis pathway. The results showed that caspase-3 and caspase-9 were significantly upregulated after ARE treatment in A549 cells. The Bax mRNA expression levels were increased in the ARE groups of the experiment. However, compared with the control group, the Bcl-2 mRNA expression level was obviously decreased. As shown in Figures 4(a)–4(d), the possible mechanisms by which ARE induces apoptosis in A549 cells can be summarized. Mitochondria apoptosis pathway plays a vital role in the ARE-induced damage effect.

### 3.5. ARE-Induced ROS Generation Reduced the Concentration of Antioxidant Enzymes in Cells

**Figure 5:**
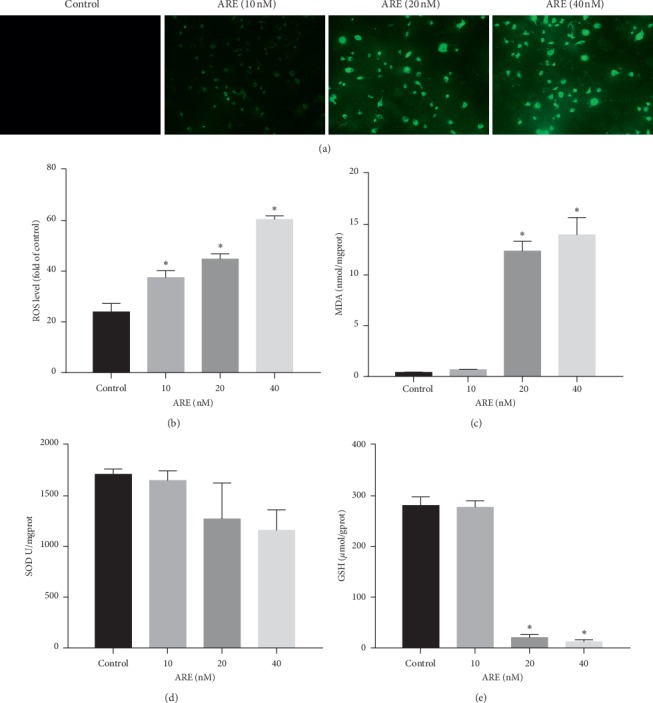
Effects of ARE on intracellular ROS and related antioxidant enzyme. A549 cells were pretreated with ARE (10–40 nM) for 24 h. (a) ROS levels were observed under the fluorescence microscope and then quantitatively analyzed by flow cytometry using DCFH-DA. (b) Quantitative analysis of ROS staining. (c) ARE treatment increased the level of MDA in A549 cells. (d), (e) ARE reduced the activity of GSH and SOD in A549 cells. The results are expressed as mean ± SD (*n* = 3). ^*∗*^*P* < 0.05 versus the control group.

The flow cytometry analysis and DCFH-DA fluorescence assay were performed to check the ROS levels of cells with green fluorescence. ROS levels in A549 cells increased after ARE treatment in a dose-dependent manner (Figures 5(a) and 5(b)). To test the antioxidant capacity of A549 cells, we examined MDA levels, as well as the activity of SOD and GSH. The level of MDA is an important parameter reflecting the potential antioxidant capacity of the body. ARE subjected A549 cells showed a significant increase in MDA levels in a dose-dependent manner (Figure 5(c)). Furthermore, ARE exposure reduced the level of SOD and GSH in A549 cells in a dose-dependent manner (Figures 5(d) and 5(e)). These data suggest that ARE could augment cell oxidative stress damage by reducing the supply of endogenous antioxidants.

### 3.6. NAC Inhibited the Cell Apoptosis Effect of ARE in A549 Cells

**Figure 6:**
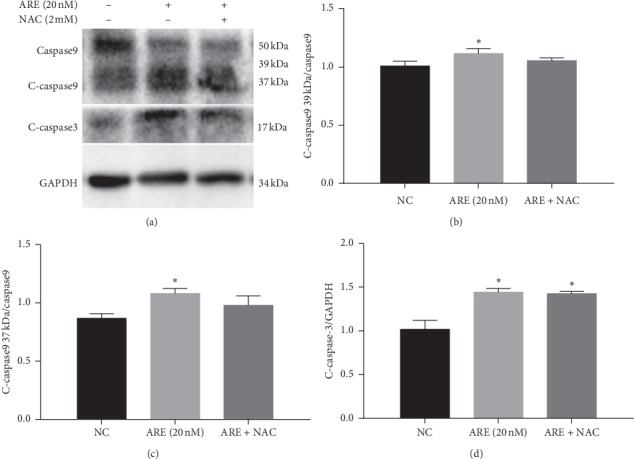
The effects of NAC on apoptotic response in A549 cells treated with ARE. Pretreatment of A549 cells with antioxidant agent and the changes of apoptosis-related proteins (Figures 6(a)–6(d)). The results are expressed as mean ± SD (*n* = 3). ^*∗*^*P* < 0.05 versus the control group.

As shown in Figures 6(a)–6(d), pretreatment of A549 cells with antioxidant agent (2 mM N-acetyl-L-cysteine, NAC) 1 h prior to ARE treatment prevented ROS generation, and cells pretreated with NAC attenuated the ROS activation and apoptotic response. These results suggest that ROS may play a key role in the initiation of apoptosis in A549 cell. Western blotting results showed that the ratio of cleaved caspase-3 and cleaved caspase-9 of A549 cells exposed to ARE significantly increased compared with the control group, but NAC decreased the cleaved caspase-3 and cleaved caspase-9 ratio compared with the ARE group.

## 4. Discussion

**Figure 7:**
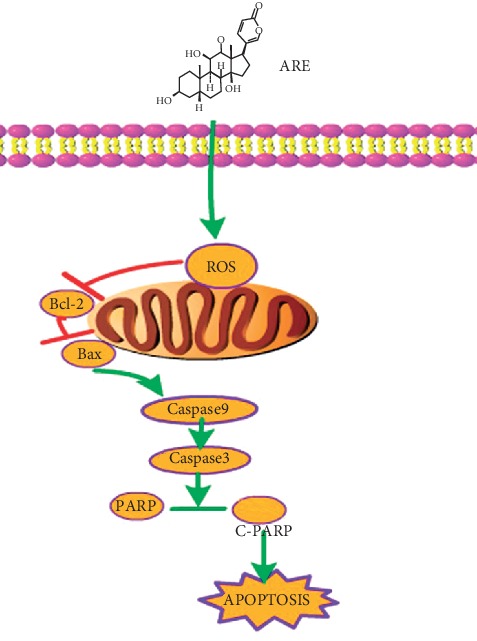
Proposed mechanisms for ARE-induced apoptosis.

Cinobufagin venom toad is a traditional Chinese herbal medicine processed from the ear glands and skin gland secretions of amphibians and reptiles of the genus *Polygonaceae*. Since ancient times, it has been widely used in China to detoxify, reduce swelling, and stop pain. ARE is an active ingredient extracted from Cinobufagin venom toad [19, 20]. There are many studies confirming that ARE has an antitumor effect. It has not been shown how ARE causes apoptosis in NSCLC. Our data confirmed that excess ROS is associated with impaired mitochondrial function and enhanced caspase 3/9 activity in ARE-induced A549 cell apoptosis, as shown in Figure 7.

In this study, we cultured A549 cells in medium containing 10% fetal bovine serum and then exposed the cells to ARE for 24 hours. ARE at 10–40 nM concentration-dependently decreased cell survival. ARE increases the LDH leakage rate in the medium and changes the morphology of A549 cells. Bax protein can facilitate apoptosis, but Bcl-2 protein can inhibit cell apoptosis, so the ratio of Bax/Bcl-2 protein states the survival of many cells including A549 cells. Bax or Bcl-2 could get command of mitochondrial permeability transition and increase cytochrome c release, thus leading to activation of caspase-9 and caspase-3 and ultimately leading to apoptosis [21, 22]. In this experiment, Bax was upregulated and Bcl-2 was downregulated, and the proportion of Bax/Bcl-2 was decreased in a dose-dependent manner after the administration of ARE to A549 cells. Caspase plays a dominant role in the entire process of apoptosis by cleavage of all kinds of specific substrates to regulate apoptosis. Our study indicates that ARE increases the expression of cleaved caspase-3, cleaved caspase-9, and PARP proteins. These results illustrate that the proapoptotic effect of ARE may be segmental due to the inactivation of cysteine protease, which is closely related to the regulation of Bax and Bcl-2 expression in A549 cells treated with ARE. Cellular damage is a complicated course involved in sundry signaling pathways and molecular mechanisms. ARE could sectionally increase cellular damage by promoting oxidative stress. Therefore, cell viability, LDH leakage, and apoptosis in A549 cells cannot be restored to normal levels after ARE treatment.

Hence, the removing of large amounts of ROS by antioxidants could be a key method to inhibit ROS-induced cell damage. Antioxidants in cells eliminate most free radicals and alleviate enormous damage derived from oxidative stress [23]. Our current research proved that ARE exposure significantly fortifies the production of ROS in A549 cells, thereby increasing the level of MDA. The levels of total SOD and GSH endogenous antioxidant enzymes in A549 cells increased after ARE treatment. This research revealed that ARE may induce A549 cell damage by decreasing the concentration level of antioxidants in cells. These results showed that MMP decreased after exposure to ARE, ultimately leading to accumulation of ROS and considerable mitochondrial dysfunction. This study indicated that ARE reduced MMP, led to dysfunction in mitochondria, and increased oxidative stress, primarily by resulting in ROS production caused by reduced antioxidant concentrations.

We revealed a novel molecular mechanism by which ARE induces apoptosis in A549 cells by activating oxidative stress pathways. Our research likewise shows that ARE may have potential use as a new therapeutic agent for NSCLC.

## Data Availability

All data generated or analyzed during this study are included within the article.
